# The social brain?

**DOI:** 10.1098/rstb.2006.2003

**Published:** 2007-01-24

**Authors:** Chris D Frith

**Affiliations:** Wellcome Department of Imaging Neuroscience, Institute of Neurology, University College London12 Queen Square, London WC1N 3BG, UK

**Keywords:** social brain, perspective taking, second-order representations

## Abstract

The notion that there is a ‘social brain’ in humans specialized for social interactions has received considerable support from brain imaging and, to a lesser extent, from lesion studies. Specific roles for the various components of the social brain are beginning to emerge. For example, the amygdala attaches emotional value to faces, enabling us to recognize expressions such as fear and trustworthiness, while the posterior superior temporal sulcus predicts the end point of the complex trajectories created when agents act upon the world. It has proved more difficult to assign a role to medial prefrontal cortex, which is consistently activated when people think about mental states. I suggest that this region may have a special role in the second-order representations needed for communicative acts when we have to represent someone else's representation of our own mental state. These cognitive processes are not specifically social, since they can be applied in other domains. However, these cognitive processes have been driven to ever higher levels of sophistication by the complexities of social interaction.

## 1. The social brain

In her seminal review, Brothers (1990) proposed that there was a circumscribed set of brain regions that were dedicated to social cognition. She called this set of regions the social brain and listed amygdala, orbital frontal cortex and temporal cortex as its major components. The evidence for her proposal came largely from studies of monkeys. After lesions to the amygdala, monkeys become socially isolated (Kling & Brothers 1992) and lesions to orbital frontal cortex can also alter social behaviour (Raleigh & Steklis 1981). Neurons in the superior temporal sulcus respond to aspects of faces such as expression and gaze direction (Perrett *et al*. 1992). With the advent of brain imaging, it has become possible to study social brain in human volunteers. Brothers' conjecture has stood up well to this barrage of new evidence (e.g. Adolphs 2003). However, there have been two major additions to the list of social brain regions. First, the medial prefrontal cortex and the adjacent paracingulate cortex have been consistently implicated in studies where participants have to think about mental states (Amodio & Frith 2006). Second, a ‘mirror’ system has been found in the brain of monkeys and humans, which allows us, to some extent, to share the experiences of others (Rizzolatti & Craighero 2004). In this essay, I shall briefly review the evidence concerning the mirror system and the four specific brain regions considered to have a role in social cognition: (i) the posterior superior temporal sulcus (pSTS) and the adjacent temporo-parietal junction (TPJ), (ii) the amygdala, (iii) the temporal poles, and (iv) the medial prefrontal cortex (MPFC) and the adjacent anterior cingulate cortex (ACC). I will speculate on the precise roles of these various systems and consider to what extent their functions are specifically social.

### (a) What is the social brain for?

But first, I must consider what the social brain is for. It is the social brain that allows us to interact with other people. As with all our interactions with the world, we can do much better if we can predict what is going to happen next. The better we can predict what someone is going to do next, the more successful our interactions with that person will be. I shall argue that the function of the social brain is to enable us to make predictions during social interactions. These predictions need not be conscious and deliberated. For example, classical Pavlovian conditioning allows us to anticipate what will happen after a conditioned stimulus. Such basic conditioning has social relevance if the conditioned stimulus is a face with a certain expression.

### (b) Prediction in social interactions

Perhaps the most important attribute of the social brain is that it allows us to make predictions about people's actions on the basis of their mental states. This assumption that behaviour is caused by mental states has been called taking an ‘intentional stance’ (Dennett 1987) or ‘having a theory of mind’ (Premack & Woodruff 1978). The largely automatic process by which we ‘read’ the mental states of others is called mentalizing.

There are many different types of mental states that can affect our behaviour. There are long-term dispositions: one person may be trustworthy while another is unreliable. There are short-term emotional states like fear and anger. There are desires like thirst which lead to specific goal-directed behaviours. There are the beliefs that we have about the world which determine our behaviour even when they are false. For example, I will look in my bookcase for a book that is not there if someone has borrowed it without telling me. Finally, there is the rather special intention to communicate with others, and the associated ability to recognize that certain behaviours are communicative.

## 2. The role of the amygdala

One of the unexpected results from early brain imaging studies was the fragmentation of emotion. There is no single brain system dedicated to emotion. Rather, each emotion has its own specific system. For example, fear is associated with activity in the amygdala (Morris *et al*. 1998), while disgust is associated with activity in the insular (Phillips *et al*. 1997). In these examples, the activity was elicited, not by the emotion directly, but by observing the expression of the emotion in a face. Thus, through its role in recognizing expressions such as fear, the amygdala has a role in social interactions.

### (a) Prejudice

But this is not its only role. The amygdala is also activated by presentation of faces rated as untrustworthy (Winston *et al*. 2002). This is an example of prejudice since the faces were of people unknown to the participants in the experiment. Race prejudice has been studied in a number of imaging paradigms and amygdala activation has been consistently found in association with the unconscious fear that is elicited by viewing the face of someone from another race. When white Americans were shown the faces of unknown black Americans, activity was observed in the amygdala (Phelps *et al*. 2000). The magnitude of the activity in the amygdala correlated with implicit measures of race prejudice. However, amygdala damage does not remove race prejudice (Phelps *et al*. 2003), and amygdala response magnitude does not correlate with explicit measures of race prejudice. Our consciously held attitudes about race are often at variance with our implicit prejudices and there is evidence that we try to suppress these rapid automatic responses. The amygdala response to black faces was reduced when the faces were presented for 525 ms rather than 30 ms and, associated with this reduction, there was increased activity in areas of frontal cortex concerned with control and regulation (Cunningham *et al*. 2004).

### (b) Prejudice and conditioning

Race prejudice is an example of stereotyping: associating mental attributes with a group of people and then applying this prejudice to individual members of that group. The amygdala is involved in this process owing to its role in fear conditioning. Extensive research with animals has shown that the amygdala is part of a system that learns to associate value with stimuli (Dolan 2002), whether or not these stimuli are social (LeDoux 2000). This system operates on both positive and negative values. For example, the amygdala responds to objects that elicit fear owing to their association with punishment (negative value), but the amygdala also responds to objects associated with food and sex (positive value).

In the experiments on race prejudice, the amygdala is responding to black faces in the same way as it responds to any object that has acquired a conditioned fear response (Buchel *et al*. 1998). The role of the amygdala in recognizing expressions of fear most probably has the same origin. A fearful expression is a signal (the conditioned stimulus) that there is something fearful near at hand (the unconditioned stimulus), so that a fearful face will eventually elicit a fear response. The amygdala is involved in social cognition owing to its role in associating the value (positive or negative) with individual objects and classes of object. This system applies to people just as it does to objects. Our long-term prejudices about individuals and groups are built up through a conditioning process involving the amygdala, but this process is not specifically social.

## 3. Temporal poles

### (a) Social scripts

Through experience, we build up a rich store of knowledge about the world (Schank & Abelson 1977) that is important for our ability to mentalize. We learn facts about specific people: what they look like, where they live, whether they are trustworthy and so on. We also learn facts about social situations: the moment-to-moment changes in behaviour appropriate to the situations in which people frequently find themselves and also how feelings and dispositions affect the behaviour of people in these situations. Damage to the temporal poles can impair the ability to use this knowledge (Funnell 2001). This observation is consistent with the suggestion that the temporal poles are convergence zones, where simpler features from different modalities are brought together to define, by their conjunction, unique individuals and situations (Damasio *et al*. 2004). Through this convergence of information, our understanding of an object can be modified by the context in which it appears (Ganis & Kutas 2003). These processes instantiated in the temporal poles are important for mentalizing. They allow us to apply our general knowledge about social situations to the situation that currently confronts us. They specify the kinds of thoughts and feelings most likely to occur in a particular context, e.g. the pride or embarrassment that we have felt or observed in similar situations in the past. But, of course, situations are never exactly repeated. There is much to be learned by observing the moment-to-moment changes in expression and behaviour in the person we are interacting with. This is the role for the brain's mirror system.

## 4. The brain's mirror system

### (a) A Bayesian approach to mentalizing

Our social brain has two problems to solve. First, it must read the mental state of the person we are interacting with. Second, it must make predictions about future behaviour on the basis of that mental state. From a Bayesian perspective, these two problems are not independent. The error in my prediction of future behaviour indicates how good my reading of the mental state was and enables me to make a better estimate of that mental state. In principle, the same mechanism can be used for reading mental states as for reading hidden states of the world outside the social domain. For example, when I reach for a coffee pot, I have to estimate how heavy it is. On the basis of this estimate, I can initiate the appropriate grasping behaviour and predict the consequences of my action. If my estimation of the hidden state of the coffee pot is wrong, my prediction will be incorrect. For example, if the pot is lighter than I expected, then my hand will move up faster than I expected. This error tells me that the coffee pot is lighter. Wolpert *et al*. (2003) have outlined how such an action system could provide the basis for reading the hidden intentions of others during action observation (see also Wilson & Knoblich 2005).

One problem for the Bayesian mechanism I have outlined is for it to get started. Where does the initial estimate of mental state come from? I suggest that this problem can be solved by the brain's mirror system. Since Gallese (2007) will be discussing this system in detail in his contribution to this issue, my comments will be brief and will emphasize my particular view.

### (b) Mirroring emotions and actions

The idea that there is a mirror system in the brain arises from the observation that the same brain areas are activated when we observe another person experiencing an emotion as when we experience the same emotion ourselves (e.g. Wicker *et al*. 2003). The brain's mirror system is engaged by actions as well as emotions and, indeed, it was this aspect of the system that was first identified (for a recent review, see Rizzolatti & Craighero 2004). Motor areas of the brain become active when we observe others moving, and also we tend to imitate the movements of others automatically (Chartrand & Bargh 1999), even when this interferes with our own actions (Kilner *et al*. 2003). The mirror system also operates for touch and for pain. Somatosensory brain regions are activated when we see someone else being touched (Keysers *et al*. 2004; Blakemore *et al*. 2005). Pain areas in the brain become active when we see someone receiving a painful stimulus (Morrison *et al*. 2004; Jackson *et al*. 2005) or even when a symbolic cue tells us that someone is receiving pain (Singer *et al*. 2004). These mirror effects can occur for auditory as well as visual cues (Kohler *et al*. 2002).

The brain's mirror system is not tied to any particular brain region. The location of the activation will depend upon what is being observed. Underpinning the mirror system, there must be some rather general mechanism by which sensory or symbolic cues can be converted into covert actions. One possibility is that the brain represents actions in the same way, whether perceiving them or planning them (the common coding principle; Prinz 1997). Such a representation does not specify who is performing the action and would be accessed when both perceiving and performing an action.

### (c) Contagion: a first step in mentalizing

Whatever the mechanism, the result is that actions are contagious. When we see someone smiling, we will automatically imitate that smile and feel happier ourselves. Through this mechanism, we can experience the emotional states of another person. I believe this phenomenon supplies the first step in mentalizing, i.e. the initial estimate of the mental state of the person we are interacting with. However, experiencing the same emotion as another is only the first step. It will not necessarily reveal the cause of the emotion. If we know that someone is afraid, we might predict that they will run, but we cannot predict where they will run unless we know what they are afraid of. Likewise, covertly performing the same movement as another is not sufficient to infer the goals and intentions behind that movement. Furthermore, as Mitchell *et al*. (2006) point out, while the mirror system is ideally suited for tracking the continually changing states of emotion and intention of the other, it can tell us nothing about the stable attitudes and predilections of the other, which are also important determinants of behaviour.

## 5. The role of posterior superior temporal sulcus/temporo-parietal junction

Through the resonance of our brain's mirror system, we might know that someone is afraid because we are sharing their experience. But how do we know what they are afraid of? One way to discover the cause of their fear is to observe where they are looking. The region of the brain at the pSTS and the adjacent TPJ is a prime candidate for this process.

### (a) Predicting movement trajectories

This region is activated when participants observe someone moving their eyes (e.g. Pelphrey *et al*. 2005) and this activity is modulated by the context in which the eye movement occurs. For example, more activity is elicited in pSTS if the actor moves her eyes away from, rather than towards, a flashing target (Pelphrey *et al*. 2004*a*,*b*). Similar effects are found when participants observe someone making reaching movements (Pelphrey *et al*. 2004*a*,*b*). One possibility is that pSTS is concerned with predicting the trajectory of movements and that greater activity is associated with prediction errors, i.e. when the movement is unexpected. For example, Saxe *et al*. (2004) showed participants a video in which an actor walked across a room. On some trials, the actor was hidden behind a bookcase. When the actor paused behind the bookcase, so that he emerged later than expected, greater activity was seen in pSTS.

But is this prediction system dedicated solely to the prediction of biological movements? Observing two balls that move in mathematically defined trajectories with no specifically biological appearance will elicit activity in pSTS as long as they appear to be interacting (Schultz *et al*. 2004, 2005). There is evidence that pSTS is involved in predicting complex movement trajectories of any kind (reviewed in Kawawaki *et al*. 2006). Perhaps the trajectory to be predicted needs to be complex, but not specifically biological to elicit activity in pSTS.

### (b) Perspective taking

By looking at someone's eyes, we can discover where they are looking, but how do we know what they can see? At the simplest level (level I perspective taking), we know that someone cannot see what we can see, as their line of sight is blocked by an obstacle. At a more complex level (level II perspective taking), we know that people looking at the same scene from different angles will arrive at different descriptions of the scene. From my point of view, the pole might be in front of the block, while from your point of view, the pole might be to the left of the block (see Aichhorn *et al*. (2005) for a useful review of perspective taking). There have been few imaging studies of this kind of spatial perspective taking, with somewhat equivocal results. Zacks *et al*. (2003) and Aichhorn *et al*. (2005) observed activity in the TPJ when participants had to describe a scene from another viewer's perspective. However, such activity was not observed in the study of Vogeley *et al*. (2004), possibly because this study involved level I rather than level II perspective taking.

Knowing where a fearful person is looking and what they can see, given their vantage point, enables us to know what they are looking at and thus identify the cause of their fear. This ability to see the world from another's perspective enables us to realize that other people can have different knowledge from us and may have false beliefs about the world, e.g. ‘he thinks he is safe because he can't see the bear coming up behind him’. There is evidence that the TPJ has a critical and more general role in the performance of tasks that depend upon understanding that a person has a false belief about the world from both imaging (Saxe & Kanwisher 2003) and lesion studies (Apperly *et al*. 2004).

Recently, the TPJ (63,−37,20) has been shown to have a critical role in how we perceive our own body in space. Abnormal electrical activity in this area in patients can create out-of-the-body experiences, in which patients experience looking down at their own body from above. Furthermore, disruption of activity in this region with transcranial magnetic stimulation in healthy volunteers can impair performance of a task which requires the imagination of one's own body as if seen from outside (Blanke *et al*. 2005). Perspective taking has an important role in social cognition, but has a role in other domains also. The ability to imagine one's body in another position in space is important for spatial memory (e.g. Nardini *et al*. 2005) as well as for social cognition.

## 6. The role of medial prefrontal cortex

Activity in MPFC was observed in the earliest studies of mentalizing (Fletcher *et al*. 1995; Goel *et al*. 1995). These observations were subsequently confirmed with a very wide range of tasks, which required participants to think about mental states (reviewed in Amodio & Frith 2006).

### (a) Is the MPFC really necessary for mentalizing?

There are, however, some unresolved problems for the interpretation of these results. First, it is not clear whether this region needs to be intact for successful performance of mentalizing tasks. On one hand, several group studies have shown that patients with damage to prefrontal cortex perform badly on mentalizing tasks and that this impairment is independent of problems with traditional executive tasks (Rowe *et al*. 2001; Stuss *et al*. 2001; Gregory *et al*. 2002). On the other hand, there is a report of a patient with damage restricted to MPFC who was not impaired on performance of mentalizing tasks (Bird *et al*. 2004). Second, there is an observation that activation of MPFC is often observed during rest or low demand tasks in comparison to high demand tasks. As a result, while activity may be seen in MPFC when mentalizing is compared with a control task (such as reasoning about physical causality), this is not always the case when mentalizing is contrasted with rest. One possible explanation for this phenomenon is that during ‘rest’ or low demand tasks, participants frequently indulge in mentalizing, thinking, for example, about why they volunteered to take part in the study or what might be the real motives of the experimenter (see Amodio & Frith (2006) for a discussion of this problem).

### (b) Which kinds of task activate MPFC?

At least, three categories of task elicit activity in MPFC and they are as follows: (i) *Mentalizing* tasks in which participants have to understand the behaviour of characters in terms of their mental states. These tasks typically involve false beliefs and can be presented as stories or cartoons (e.g. Gallagher *et al*. 2000). However, MPFC is also active when participants engage in real-time social interactions (e.g. McCabe *et al*. 2001) or even when they simply observe social interactions (Iacoboni *et al*. 2004). These tasks presumably involve predicting people's behaviour in terms of their current beliefs and intentions. (ii) *Person perception* tasks in which participants answer questions about long-term dispositions and attitudes. These can be general (e.g. Can people be dependable?—Mitchell *et al*. 2002) or specific (e.g. Is your mother talkative?—Schmitz *et al*. 2004) and need not apply only to people (e.g. Can dogs be dependable?—Mitchell *et al*. 2005). (iii) *Self-perception* tasks in which participants answer questions about their own long-term dispositions (e.g. Are you talkative?—Kelley *et al*. 2002) or about their current feelings (Does this photo make you feel pleasant?—Ochsner *et al*. 2004). These three kinds of task have in common the need to think about mental states. These can be short-term or long-term mental states and can be of the self or another.

### (c) Location of the mentalizing region within MPFC

There is little evidence for any systematic differences in the location of the activity associated with these three kinds of task. The activity is located in a diffuse region (paracingulate cortex) on the border of anterior cingulate cortex (BA 32) and medial prefrontal cortex proper (BA 10). This region has been labelled the ‘emotional’ region of MPFC and is more anterior and inferior to the region labelled ‘cognitive’ (Steele & Lawrie 2004). In anatomical terms, it can be labelled anterior rostral MPFC (Amodio & Frith 2006). A meta-analysis of studies where activity has been observed in area 10 (Gilbert *et al*. 2006) shows that activity associated with mentalizing tasks is medial rather than lateral and is posterior to the activity associated with multi-task coordination, which is observed at the frontal pole.

### (d) A role for anterior rostral MPFC?

There is, however, strong evidence for a different role for anterior rostral MPFC in comparison to the adjacent regions of medial prefrontal cortex. For example, in the study of Mitchell *et al*. (2006), participants were told about two target individuals who were described as having liberal or conservative views. They were then asked to predict the feelings and attitudes of these two individuals in various situations (e.g. ‘would he enjoy having a roommate from a different country?’). The results show a different pattern when thinking about a similar or a dissimilar other. Thinking about similar others was associated with activity in ventral mPFC (18,57,9: in the region labelled anterior rostral MFC in Amodio & Frith 2006), while thinking about a dissimilar other was associated with activity in a more dorsal region of mPFC (−9,45,42: posterior rostral MFC).

Previous studies had also observed distinctions between anterior and posterior rostral MPFC. Walter *et al*. (2004) asked participants to make inferences about private intentions (changing a broken light bulb in order to read a book) in contrast to communicative intentions (showing someone a map in order to ask the way). Thinking about communicative intentions activated a more ventral region (−3,54,15: arMPFC) than thinking about private intentions. Grezes *et al*. (2004*a*) asked participants to infer whether the movements associated with the lifting of a box were intended to be deceptive since the actor was pretending that the box was heavier than it really was. Movements thought to be deceptive activated arMPFC (−8,42,20). In another experiment (Grezes *et al*. 2004*b*), the participants observed movements, which sometimes included unexpected adjustments, because the box being picked up was lighter than the actor expected. Observing these unexpected adjustments was associated with activity in prMPFC (2,26,52).

These results suggest that arMPFC has a special role in handling communicative intentions. This is a more complex process than simply thinking about intentions, since we have to recognize that the communicator is also thinking about our mental state. This involves a second-order representation of mental state. We have to represent the communicator's representation of our mental state. This is a form of triadic social interaction, such as joint attention, that Saxe (2006) also associates with dorsal MPFC. In relation to the observations of Mitchell *et al*. (2006), when we think about the mental states of people with similar attitudes to ourselves, perhaps we automatically think in terms of our shared view of the world.

Such second-order representations are not necessary when thinking about another person's private intentions or their beliefs about the weight of a box. In these examples, we are simply predicting the outcomes of actions. Several studies have investigated the prediction and monitoring processes associated with the selection of action. Walton *et al*. (2004) observed activity in the prMFC when participants monitored the outcome of actions that were self-selected. Knutson *et al*. (2005) reported that the activity in the prMPFC was correlated with trial-by-trial variations in the anticipated probability of monetary gain. In research by Coricelli *et al*. (2005), a similar region of prMPFC activity was associated with regret, i.e. discovering that an unselected action would have led to a better outcome. Finally, Brown & Braver (2001) reported that prMFC activation was associated with prediction of the probability of error.

These results all suggest that this region is concerned with predicting the probable value of actions of the self. However, the results of EEG studies show that this region is also involved when we observe the actions of others. A negative event-related potential component arising from the MPFC is seen not only when we make an error, but also when we receive delayed error feedback (Gehring & Willoughby 2002; Luu *et al*. 2003) or observe someone else making an error (van Schie *et al*. 2004; Bates *et al*. 2005).

Inferior to the arMPFC is the orbital region. This region seems to be concerned with feelings rather than actions, particularly feelings relating to anticipated rewards and punishments. In monkeys, Padoa-Schioppa & Assad (2006) have shown that the value of offered goods is represented in orbital frontal cortex. In humans, Coricelli *et al*. (2005) found that activity in this region (−10,40,−24) was associated with anticipated regret. Again, this monitoring of feelings seems to apply to others as well as the self. Hynes *et al*. (2006) asked participants to make inferences about what other people were thinking (cognitive perspective taking) or what they were feeling (emotional perspective taking). Thinking about people's feelings was associated with activity in medial orbital cortex (18,63,−7), while perspective taking in general was associated with activity in more dorsal regions (2,59,15;−4,60,30).

## 7. The function of intellect

I have speculated about the role of various components of the social brain, but in most cases, I believe that these processes are not specifically social. The exception is the brain's mirror system.

### (a) Accessing the mirror system: the role of social variables

Activation of the mirror system seems to be a largely automatic process that is not under conscious control. But we do not mirror everything that moves and not everyone receives our empathy. Watching a moving human arm will interfere with our own movements, but this interference does not happen when we watch a moving robot arm (Kilner *et al*. 2003). This tuning of the mirror system to purposeful agents rather than machines is observed in brain activity as well as behaviour in monkeys and humans (for a review, see Tai *et al*. 2004). These results suggest that signals from an ‘agent detector’ or perhaps a ‘conspecific detector’ are needed to turn on the action mirror system.

In the case of empathy, or mirroring of emotions, the situation is more complex. How much empathy we show to conspecifics is modified by our social relationship with the object of our empathy. We feel less empathy, in terms of subjective report and brain activity, if someone who has just treated us unfairly receives pain, especially if we are male (Singer *et al*. 2006). We also show more empathy with the pain of another if we are in eye contact with them at the moment they receive the pain (Bavelas *et al*. 1986). Showing one's emotion is, in part, a communicative act (Parkinson 2005) facilitated by the presence of others. It is not yet known whether the neural correlates of empathy are also modified by communicative contact, but, in any case, the functioning of our brain's mirror system is clearly subject to exquisite modulation by social variables.

### (b) Cognition in the service of social interaction

The amygdala is concerned with conditioning, enabling emotional valence to be associated with an object. This object may often be a face, but the process also applies to objects, such as snakes, with no social connotations. The role of the temporal poles is less well understood, but here again, while the high-level concepts and the scripts for different circumstances instantiated here are of great importance for social cognition, these forms of knowledge are important for interacting with the physical as well as the mental world.

I have already suggested that the role of pSTS in processing biological motion might be a consequence of a more general role in predicting complex movement trajectories. While such trajectories are often created by biological agents, they can have other sources. Likewise, if the role of the TPJ is, as I suggest, to compute different spatial perspectives, then while this is very useful for social interactions, the process has much more general applications.

Most speculative and least well specified of all the suggestions in this essay is the role of anterior rostral MPFC. In terms of anatomy, there is some evidence that this region has shown disproportionate expansion in recent evolution (Semendeferi *et al*. 2001). The evidence for phylogenetic expansion of the social brain is discussed in some detail by Dunbar in this issue (Dunbar & Shultz 2007). In cognitive terms, this region of medial prefrontal cortex seems to be activated in scenarios involving communicative intent. Such scenarios involve second-order representations of mental states, since, to understand your attempt to communicate with me, I have to represent your representation of my mental state. This example concerns a high-level social interaction, but such second-order representations need not have social content. For example, second-order representations may have something in common with the higher-order thoughts that may be necessary for conscious experience (Rosenthal 2005). However, these are essentially general purpose operations that can be applied to any domain.

### (c) The needs of social interaction drive cognition

However, although these mechanisms are not specifically social, they have been strongly influenced by the needs of social interactions. Humans are not only much more sophisticated in their social interactions than other animals; they are also much cleverer and more inventive in many domains that are not social, for example in the use of materials for making novel objects. This is the critical point that Nick Humphrey (1976) made in his paper: these cognitive functions have evolved to their high level because they have been driven by the complexities of social living. In order to avoid untrustworthy faces, we need a visual system that can process the subtle visual features which reveal personality. To discover what someone is interested in, we need to be able to compute what someone on the other side of the room can see. We need to acquire and store the complex scripts that enable us to behave appropriately at discussion meetings. And, above all, we need to be able to represent what other people think about us so that we can use such venues to enhance all our reputations.
